# Communication-Efficient Federated Learning with Dual-Sided Sparse Aggregation for Edge Sensing Systems

**DOI:** 10.3390/s26092885

**Published:** 2026-05-05

**Authors:** He Zhao, Jingwei Li

**Affiliations:** School of Telecommunications Engineering, Xidian University, Xi’an 710071, China; zhaohe1@stu.xidian.edu.cn

**Keywords:** federated learning, non-IID data, model pruning, FedProx regularization, communication efficiency

## Abstract

Distributed edge sensing systems, such as IoT monitoring nodes, wearable devices, and camera-based sensing terminals, continuously generate privacy-sensitive data that are costly to transmit to a central server. Federated learning (FL) provides a promising solution for collaborative model training without raw-data sharing; however, its practical deployment in edge sensing systems is challenged by non-IID local observations, limited uplink/downlink resources, and restricted on-device computation. To address these issues, this paper proposes a Dual-Sided Sparse Aggregation (DSSA) mechanism integrated with FedProx for resource-constrained edge sensing environments. In the proposed framework, the server prunes the global model after each communication round and transmits only the retained parameters, while clients update the complementary parameters and upload sparse local gradients. This fixed-structure sparse training strategy reduces bidirectional communication overhead and local computation cost, while FedProx improves robustness under heterogeneous data distributions. Experiments on CIFAR-10 and SVHN with varying non-IID degrees, pruning ratios, and hyperparameter settings show that the proposed method achieves a favorable resource-performance trade-off, reducing communication cost by up to 73.0% and computation cost by up to 34.9% while maintaining competitive accuracy. Under controlled benchmark settings, the proposed method demonstrates substantial resource savings compared with FedAvg, particularly in mildly heterogeneous scenarios, indicating a favorable benchmark-level resource-performance trade-off for resource-constrained edge sensing scenarios under the evaluated settings.

## 1. Introduction

Distributed edge sensing systems are increasingly deployed in applications such as environmental monitoring, wearable health sensing, intelligent transportation, industrial Internet of Things (IoT), and camera-based infrastructure surveillance. These systems consist of large numbers of geographically distributed sensing nodes that continuously generate heterogeneous and often privacy-sensitive data. In many practical scenarios, transmitting such raw data to a central server is either prohibitively costly or undesirable due to communication overhead, latency constraints, and privacy concerns. This creates a fundamental challenge for edge sensing systems: how to enable collaborative intelligence across distributed devices while avoiding centralized data collection.

Federated learning (FL), first introduced by McMahan et al. [1], provides a promising solution to this challenge by enabling multiple sensing devices or edge clients to jointly train a shared model without exchanging raw data [2]. By keeping local observations on-device and communicating only model updates, FL has attracted considerable attention in privacy-sensitive sensing applications, including healthcare monitoring [3], mobile and wearable systems [4], and intelligent service platforms [5]. This makes FL particularly appealing for distributed sensing environments, where data are naturally decentralized and device-side resource constraints must be carefully considered.

Despite these advantages, FL still faces two major challenges in practical deployment: the inherently non-IID nature of client data and the high communication and computational costs associated with frequent model exchanges. These limitations often result in slow convergence and inefficient resource utilization, particularly in large-scale deployments [6,7]. The non-IID nature of client data causes client drift, where local models diverge from the global optimum and degrade global model performance [6]. Meanwhile, repeated communication of model parameters or gradients can impose a substantial burden on edge systems, especially when devices operate under constrained transmission and energy budgets. On the computational side, resource-constrained devices such as smartphones and IoT nodes may struggle to perform repeated local training on deep neural networks, leading to increased energy consumption, delayed updates, and the presence of stragglers that further slow down global convergence [7].

To address these challenges, several approaches have been proposed. Zhao et al. [8] introduced data sharing through a small global dataset to mitigate data heterogeneity, although this weakens the privacy-preserving nature of FL. Optimization-based methods such as FedProx [9], FedNova [10], and SCAFFOLD [11] improve convergence under heterogeneous data distributions. However, FedProx may require careful parameter tuning, FedNova primarily focuses on optimization inconsistency rather than communication reduction, and SCAFFOLD introduces additional control variates that increase algorithmic and computational complexity.

Model pruning and sparsification methods have shown particular promise for reducing communication overhead in FL. PruneFL [12] reduced communication cost through adaptive width reduction while preserving most of the baseline accuracy under IID settings. The Complement Sparsification (CS) method [13] further explored complementary pruning at both the server and client sides, but sparse training may still involve substantial forward and backward computation. More recently, federated transfer learning (FTL) has emerged as another promising direction [14]. By leveraging pre-trained models, FTL can improve learning performance under heterogeneous data without requiring substantial architectural redesign [15]. Nevertheless, reliance on pre-training may introduce additional preparation cost and may limit adaptability in dynamic environments.

Beyond optimization and compression-based solutions, emerging paradigms such as reinforcement learning [16] and meta-learning [17] have also been explored to improve FL performance under non-IID data. These methods adaptively guide optimization or learn task-specific initialization strategies, but they often rely on more sophisticated learning frameworks and higher computational complexity, which can limit their applicability in resource-constrained sensing environments.

Taken together, although FL research has made substantial progress, existing solutions still involve trade-offs among communication efficiency, computational feasibility, and robustness to non-IID data. This motivates the development of lightweight FL strategies tailored to the practical constraints of distributed edge sensing systems.

In this work, we propose a Dual-Sided Sparse Aggregation (DSSA) framework integrated with FedProx for resource-constrained edge sensing systems. The proposed framework is designed to reduce communication overhead, alleviate local computational burden, and improve robustness under heterogeneous data distributions. DSSA adopts a lightweight and structure-fixed pruning strategy. During the initial rounds, standard full-model training is performed to establish a stable starting point. The server then prunes less significant weights according to their magnitudes, generating a sparse model together with a pruning mask. In subsequent rounds, clients receive the sparse model and update only the complementary pruned weights, which reduces both local computation and the size of transmitted updates. The server aggregates these sparse updates into the global dense model, and the pruning process is repeated iteratively.

Compared with existing communication-efficient FL methods, the proposed DSSA framework offers a communication- and computation-efficient alternative for edge sensing systems. First, unlike CS [13], which maintains fixed sparsity patterns across rounds, DSSA employs a dynamic weight rotation mechanism that enables broader parameter participation over time while preserving sparsity. Second, unlike approaches that combine sparsification with additional quantization or fine-tuning stages, DSSA avoids extra fine-tuning operations through its complementary update mechanism, thereby reducing the burden on resource-limited clients. Third, by preserving the retained weight structure while updating complementary parameters, DSSA provides a stable and lightweight alternative to more aggressive gradient compression strategies. To further improve robustness in heterogeneous sensing environments, we integrate FedProx into the proposed framework. Experimental results show that DSSA can substantially reduce bidirectional communication and local computation, demonstrating a favorable resource-performance trade-off under the tested settings with moderate performance degradation. These results suggest that, based on the benchmark evaluations conducted in this study, DSSA offers a promising FL alternative for distributed edge sensing systems where communication and computation resources are constrained.

## 2. System Model

### 2.1. FL Model

**Figure 1:**
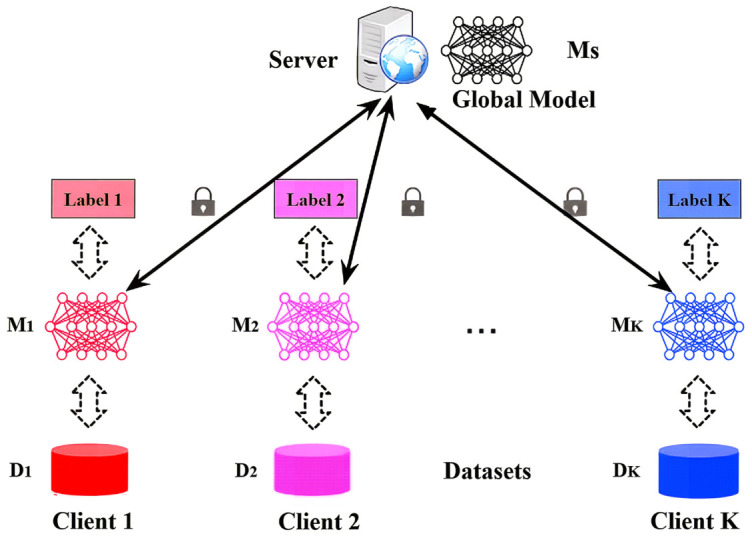
Federated learning model demonstration.

Federated Averaging (FedAvg) [1] is a foundational algorithm in federated learning, enabling model training across decentralized clients without exposing private data, as illustrated in Figure 1. A central server initializes a global model and broadcasts it to clients, which then perform local training on their private datasets. The local objective of client *k* is to minimize its empirical loss:

(1) $$\min_{w}F_{k}\left(w\right)=\frac{1}{n_{k}}\sum_{i=1}^{n_{k}}\ell \left(x_{i},y_{i},w\right),$$

where *ℓ* is the loss function, $\left(x_{i},y_{i}\right)$ denotes a data sample, $n_{k}$ is the number of samples, and *w* the model parameters.

Each client updates the model through several local epochs of stochastic gradient descent (SGD) and sends the results to the server. The server then aggregates these updates via weighted averaging according to local data sizes:

(2) $$w_{t+1}=\sum_{k=1}^{K}\frac{n_{k}}{n}w_{t}^{k},$$

with the total sample size:

(3) $$n=\sum_{k=1}^{K}n_{k},$$

producing a refined global model for the next round. This process repeats until convergence.

FedAvg achieves privacy preservation by keeping raw data local and reduces communication overhead through multiple local updates per round. Nonetheless, it faces challenges under statistical heterogeneity (non-IID data) and system heterogeneity (varying client resources), motivating numerous subsequent extensions.

### 2.2. Data Distribution Model

The Dirichlet distribution is commonly used in federated learning literature to simulate non-IID data distributions among clients [9,14,15,16,18,19,20]. The probability density function of the K-dimensional Dirichlet distribution is given by:

(4) $$f\left(x_{1},\ldots ,x_{K};\alpha_{1},\ldots ,\alpha_{K}\right)=\frac{1}{B(\alpha )}\prod_{i=1}^{K}x_{i}^{\alpha_{i}-1}$$

where $x_{i}\geq 0$, $\sum_{i=1}^{K}x_{i}=1$, and B(α) is the multivariate Beta function.

The concentration parameter α (typically using a symmetric Dirichlet with $\alpha_{1}=\ldots =\alpha_{K}=\alpha$) controls the degree of data heterogeneity among clients. When α→∞, the distribution approaches uniform allocation (IID setting). As α→0, the data distribution becomes increasingly skewed, with each client potentially dominated by only a few classes (highly non-IID). Many studies [15,19,20] have utilized different α values to simulate various non-IID scenarios, typically employing α=0.1 to 1.0 for strong non-IID settings and α=10 to 100 for near-IID conditions.

### 2.3. Communication Model

In this work, communication efficiency is characterized in terms of the number of model parameters transmitted between the server and clients during each communication round. Since the objective of DSSA is to reduce the payload of model exchange, we adopt a lightweight parameter-level communication model rather than a detailed physical-layer transmission model. This abstraction is suitable for evaluating the communication burden of federated learning in resource-constrained edge sensing systems.

Let *P* denote the total number of parameters in the full global model. In conventional FL, the server broadcasts the full model to each selected client, and each client uploads a full model update or gradient vector of the same size. Therefore, the downlink and uplink communication costs per client in one round can be expressed as

(5) $$C_{\mathrm{dl}}^{\mathrm{full}}=P,\;C_{\mathrm{ul}}^{\mathrm{full}}=P,$$

where the unit is the number of transmitted parameters. If each parameter is represented using *b* bits, the corresponding communication payload in bits is given by

(6) $$S_{\mathrm{dl}}^{\mathrm{full}}=Pb,\;S_{\mathrm{ul}}^{\mathrm{full}}=Pb.$$

In DSSA, the server prunes the global model before transmission. Let $p_{s}\in \left[0,1\right]$ denote the server-side pruning ratio, so that only a fraction $\left(1-p_{s}\right)$ of the model parameters is retained and transmitted to clients. The downlink communication cost per client then becomes

(7) $$C_{\mathrm{dl}}^{\mathrm{DSSA}}=\left(1-p_{s}\right)P.$$

On the uplink, each client uploads only the complementary sparse update associated with the pruned coordinates. Let $p_{c}\in \left[0,1\right]$ denote the effective sparsity ratio of the uploaded client update. Then the uplink communication cost per client can be written as

(8) $$C_{\mathrm{ul}}^{\mathrm{DSSA}}=\left(1-p_{c}\right)P.$$

Accordingly, the corresponding payload sizes in bits are

(9) $$S_{\mathrm{dl}}^{\mathrm{DSSA}}=\left(1-p_{s}\right)Pb,\;S_{\mathrm{ul}}^{\mathrm{DSSA}}=\left(1-p_{c}\right)Pb.$$

Suppose that *K* clients participate in a communication round. The total communication payload of conventional FL is

(10) $$S_{\mathrm{total}}^{\mathrm{full}}=K\left(S_{\mathrm{dl}}^{\mathrm{full}}+S_{\mathrm{ul}}^{\mathrm{full}}\right)=2KPb,$$

whereas the total communication payload of DSSA becomes

(11) $$S_{\mathrm{total}}^{\mathrm{DSSA}}=K\left(S_{\mathrm{dl}}^{\mathrm{DSSA}}+S_{\mathrm{ul}}^{\mathrm{DSSA}}\right)=K\left[\left(1-p_{s}\right)+\left(1-p_{c}\right)\right]Pb.$$

Therefore, the communication reduction achieved by DSSA depends directly on the sparsity levels applied at the server and client sides. By transmitting only the retained global parameters on the downlink and only complementary sparse updates on the uplink, DSSA substantially reduces the bidirectional communication burden compared with dense model exchange. This communication model is consistent with the objective of our method and provides a clear basis for reporting communication cost in terms of transmitted model size.

## 3. Algorithmic Description

### 3.1. FedProx

While FedAvg is effective in homogeneous environments, it often suffers from client drift under statistical heterogeneity (non-IID data) and struggles with system heterogeneity, as it requires all clients to perform a fixed number of local updates before aggregation. FedProx [9] addresses these issues by modifying the local optimization objective. Instead of minimizing only the local empirical risk $F_{k}\left(x\right)$, each client solves:

(12) $$\min_{x}h_{k}\left(x,x_{t}\right)=F_{k}\left(x\right)+\frac{\mu }{2}{\left|x-x_{t}\right|}^{2},$$

where $x_{t}$ is the global model from the server and μ controls the strength of the proximal term. This additional term $\frac{\mu }{2}{\left|w-w^{t}\right|}^{2}$ acts as a regularizer that anchors local updates to the global model, reducing divergence in non-IID settings.

Moreover, FedProx accommodates system heterogeneity by allowing partial work: clients can perform variable numbers of local iterations according to their computational resources, while the proximal term ensures their updates remain compatible with global aggregation. This unified treatment of statistical and system heterogeneity yields improved stability, convergence, and accuracy compared with FedAvg, making FedProx a more practical choice for real-world federated learning deployments.

### 3.2. DSSA

The DSSA algorithm is an innovative framework designed to enhance federated learning efficiency by addressing non-IID data distribution and resource constraints. DSSA is a sparsification framework that can be combined with standard FedAvg or with FedProx. In this paper, we focus on its integration with FedProx to improve robustness under heterogeneous data distributions. DSSA introduces a bidirectional sparsity mechanism to reduce communication and computational overhead while maintaining model performance.

DSSA operates through coordinated server-client interactions. On the server side, the global model undergoes structured pruning after each training round, where only parameters with significant magnitudes are retained for distribution to clients. This reduces downstream communication costs. On the client side, devices focus exclusively on updating the complementary weights and upload only these sparse updates. This approach eliminates the need for dynamic pruning adjustments during training, ensuring stability while minimizing resource consumption.

The algorithm’s core innovation lies in its per-round dynamic pruning masks and complementary weight assignment mechanism. This design enables DSSA to achieve significant communication savings while effectively avoiding the computational overhead associated with adaptive pruning methods. Experimental results on non-IID benchmarks demonstrate that DSSA maintains competitive accuracy with 40–60% reduced communication costs compared to standard federated learning approaches, making it particularly suitable for resource-constrained edge computing environments.

The pruning mechanism in DSSA follows a global magnitude pruning strategy, where weights across all layers are concatenated and pruned based on their absolute values, rather than applying a uniform sparsity ratio per layer. Only weight parameters are pruned; bias terms remain unchanged and are always transmitted and updated, as biases typically constitute a negligible fraction of total parameters and pruning them yields minimal communication savings while potentially degrading model performance. The pruning mask is recomputed at the beginning of every sparse communication round based on the current dense global model, rather than being inherited from the previous round or applied to the pruned model. This ensures that the most significant weights are always selected for retention according to the latest model state. For sparsity control, a global top-k selection is used: a target sparsity ratio *p* is specified, and the top k=(1−p)×N weights with the largest absolute values are retained, where N denotes the total number of weights across all layers. In cases where multiple weights share equal magnitude values at the pruning threshold, the selection follows a deterministic tie-breaking rule based on the original parameter order, ensuring reproducibility without introducing randomness. Regarding the sparse update mechanism, during local training, only the coordinates that were pruned are trainable, while the retained weights remain frozen. Clients update only these complementary weights and upload their sparse updates, which correspond exclusively to the previously pruned parameters.

### 3.3. FedProx with DSSA

Federated learning in edge sensing systems must simultaneously cope with data heterogeneity, limited device-side computation, and repeated communication overhead. FedProx improves robustness under non-IID data by regularizing local updates around the current global model, whereas DSSA reduces communication and computation cost through dual-sided sparsification. Motivated by their complementary strengths, we integrate FedProx with DSSA to obtain a lightweight FL framework that may be better suited to resource-constrained sensing environments. The overall server-side and client-side procedures are summarized in Algorithms 1 and 2.
**Algorithm 1** Server-side Execution of FedProx-DSSA  1: Initialize global model $w_{0}$  2: Set server sparsity ratio $p_{s}$, aggregation scaling factor $\eta^{\prime }$, and proximal coefficient μ  3: **for** round t=0,1,…,T−1 **do**  4:  **if** t=0 **then**  5:   **for** each client *n* in parallel **do**  6:    $\Delta_{n}\leftarrow \mathrm{ClientUpdate}\left(w_{t},\mu ,\mathrm{full}\_\mathrm{mask}\right)$  7:   **end for**  8:   Aggregate client updates:$$w_{t+1}\leftarrow \sum_{n=1}^{N}\frac{|D_{n}|}{\left|D\right|}\Delta_{n}$$  9:  **else**10:   ${\tilde{w}}_{t},\mathrm{mask}\leftarrow \mathrm{Prune}\left(w_{t},p_{s}\right)$11:   Broadcast ${\tilde{w}}_{t}$ and mask to all selected clients12:   **for** each client *n* in parallel **do**13:    $\Delta_{n}\leftarrow \mathrm{ClientUpdate}\left({\tilde{w}}_{t},\mu ,\mathrm{mask}\right)$14:   **end for**15:   Aggregate sparse complementary updates:$$w_{t+1}\leftarrow {\tilde{w}}_{t}+\eta^{\prime }\sum_{n=1}^{N}\frac{|D_{n}|}{\left|D\right|}\Delta_{n}$$16:  **end if**17: **end for**

**Algorithm 2** ClientUpdate (w,μ,mask)  1: Initialize local model θ←w  2: **for** each local batch *b* **do**  3:  Compute proximal gradient:$g\leftarrow \nabla F_{n}\left(\theta ;b\right)+\mu \left(\theta -w\right)$  4:  Update local model:θ←θ−ηg  5: **end for**  6: Extract complementary sparse update:Δ←(θ−w)⊙(1−mask)  7: **return** 
Δ

The key idea of FedProx-DSSA is to combine proximal regularization with structured complementary sparsification. At the beginning of each sparse round, the server prunes the current global model according to weight magnitude and broadcasts the retained model parameters together with the associated mask. Each client then performs local optimization using the FedProx objective

(13) $$\min_{\theta }\;F_{n}\left(\theta \right)+\frac{\mu }{2}{∥\theta -{\tilde{w}}_{t}∥}^{2},$$

where ${\tilde{w}}_{t}$ denotes the pruned global model received from the server. After local training, the client does not transmit the full model difference. Instead, it uploads only the complementary sparse update on the pruned positions, namely

(14) $$\Delta_{n}^{t}=\left(\theta_{n}^{t}-{\tilde{w}}_{t}\right)⊙\left(1-\mathrm{mask}\right).$$

The server then aggregates these sparse updates and injects them back into the pruned global model:

(15) $$w_{t+1}={\tilde{w}}_{t}+\eta^{\prime }\sum_{n=1}^{N}\frac{|D_{n}|}{\left|D\right|}\Delta_{n}^{t}.$$

This update rule can be interpreted as a sparse perturbation of a standard FedProx/FedAvg-style aggregation step. In particular, when the pruning distortion is moderate, the pruned model ${\tilde{w}}_{t}$ remains close to the dense global model $w_{t}$. Meanwhile, the uploaded update $\Delta_{n}^{t}$ captures only the complementary coordinates rather than the full local model displacement. Therefore, the proposed aggregation should not be viewed as strictly identical to FedAvg, but rather as an approximation in which only a selected subset of coordinates is corrected at each round. From this perspective, DSSA preserves the main global optimization trajectory while reducing the amount of communication and local computation. The dynamic re-pruning mechanism further allows different coordinates to participate across rounds, which helps avoid permanently freezing the same subset of parameters.

The resource-saving effect of FedProx-DSSA can be understood from both communication and computation perspectives. Let $p_{s}\in \left[0,1\right]$ denote the server-side pruning ratio. Then only approximately $\left(1-p_{s}\right)$ of the model parameters are transmitted from the server to the clients in each sparse round. On the uplink, each client sends only the complementary sparse update defined in (14), whose size is proportional to the number of active complementary coordinates. As a result, the bidirectional communication payload is substantially reduced compared with dense model exchange.

On the computation side, local training is not performed on a fixed sparse model. Instead, in each communication round, the server prunes the current dense global model by identifying weights with small magnitudes and setting them to zero, retaining only the large-magnitude weights. The resulting sparse model is transmitted to clients, who then train exclusively on the pruned coordinates while keeping the retained large-magnitude weights frozen. Because the pruning mask is recomputed every round from the latest dense global model, the set of weights that are considered small and thus trainable can change over time: previously pruned weights may grow and become retained in future rounds, while previously retained weights may shrink and become trainable. This dynamic sparsity reduces the number of active parameters in forward and backward propagation compared to dense training, while still allowing the full parameter space to be gradually updated across rounds. The additional proximal term in (13) introduces only a lightweight quadratic regularization term and therefore contributes negligible extra overhead in practice. Overall, within the scope of our experimental evaluation, FedProx-DSSA provides a favorable trade-off between robustness to heterogeneous data and resource efficiency, making it suitable for FL deployment in edge sensing systems with constrained computation and communication budgets.

### 3.4. Mechanism Discussion

While DSSA reduces communication and computation overhead through bidirectional sparsity, its aggregation rule constitutes an approximation to standard dense federated averaging. Understanding the nature of this approximation and its reliability is essential for proper interpretation of the empirical results.

In standard FedAvg, the server aggregates full client model updates and computes the new global model as $w_{t+1}=w_{t}+\frac{1}{M}\sum_{i=1}^{M}\Delta w_{i}^{\left(t\right)}$, where $\Delta w_{i}^{\left(t\right)}$ represents the full parameter change from client *i*. In DSSA, after the initial warm-up rounds, the server maintains a pruned dense model $w_{t}^{\mathrm{pruned}}$, where a subset of weights is retained and the remaining coordinates are set to zero. Clients receive this pruned model and train only on the complementary coordinates. The uploaded client updates thus contain only changes to these complementary parameters. The server then aggregates these sparse updates and combines them with the retained weights via:

(16) $$w_{t+1}^{\mathrm{pruned}}=w_{t}^{\mathrm{pruned}}+\eta^{\prime }\cdot \frac{1}{M}\sum_{i=1}^{M}\Delta w_{i}^{\mathrm{comp},(t)}$$

where $\Delta w_{i}^{\mathrm{comp},(t)}$ denotes the update restricted to the complementary coordinates, and $\eta^{\prime }$ is an aggregation scaling factor. This process deviates from standard dense aggregation in two fundamental ways: the retained weights are never updated directly during local training, and only a subset of coordinates participates in the aggregation. Consequently, DSSA does not exactly implement FedAvg or FedProx on the full parameter space but instead performs a constrained optimization where the update direction is projected onto the complementary subspace.

The reliability of this sparse aggregation approximation depends on several factors. First, when pruning distortion is moderate, the retained weights capture the dominant structure of the global model, ensuring that the frozen parameters already represent a reasonable solution. Second, the complementary coordinates are iteratively corrected across rounds. Although each round only updates the pruned coordinates, the pruning mask is recomputed every round based on the current dense global model. This dynamic mask rotation mechanism allows coordinates that were previously frozen to become trainable in subsequent rounds, enabling gradual refinement of the entire parameter space over time. Third, the aggregation ratio $\eta^{\prime }$ compensates for magnitude attenuation. Because clients only update the complementary coordinates, the magnitude of the aggregated update may be systematically smaller than in the dense case. Scaling this update by $\eta^{\prime }$ helps align the effective step size with that of standard aggregation.

The hyperparameter $\eta^{\prime }$ serves as a compensatory scaling factor for sparse updates. Its primary role is to compensate for the magnitude attenuation caused by updating only a subset of coordinates. However, based on our empirical observations, the optimal value of $\eta^{\prime }$ does not necessarily follow a simple monotonic relationship with sparsity or data heterogeneity, as it is jointly influenced by the norm of the sparse updates, client drift, optimizer behavior, and the distribution of trainable complementary coordinates. Therefore, rather than deriving it from a theoretical rule, we treat $\eta^{\prime }$ as a configuration-dependent empirical hyperparameter. For each configuration of sparsity and heterogeneity, we select the optimal value from the candidate set {3,5,7,10} based on empirical performance. The effectiveness of this empirically guided selection is supported by our experimental observations under the tested configurations, demonstrating that DSSA achieves competitive performance with this tuning strategy.

## 4. Results and Discussion

### 4.1. Experimental Evaluation

All experiments were conducted on an NVIDIA RTX 4090 GPU using the CIFAR-10 and SVHN dataset [21]. The neural network architecture is a Convolutional Neural Network (CNN) comprising three convolutional layers followed by two fully connected layers, processing 32×32 RGB images. This paper adopts CNNs instead of more complex neural network architectures, primarily because it focuses on the communication-computation trade-offs in federated learning under resource-constrained sensing scenarios, rather than pursuing the highest possible accuracy from a single node. Under this objective, CNNs provide a lightweight, stable, and practically deployable backbone for controlled evaluation in resource-constrained sensing settings. The two datasets are partitioned in a non-IID manner across 10 clients. The model contains approximately 1.1 million trainable parameters. All convolutional and fully connected layers include bias terms. Each convolutional layer is followed by ReLU activation and 2×2 max pooling, and no dropout is applied.

**Figure 2:**
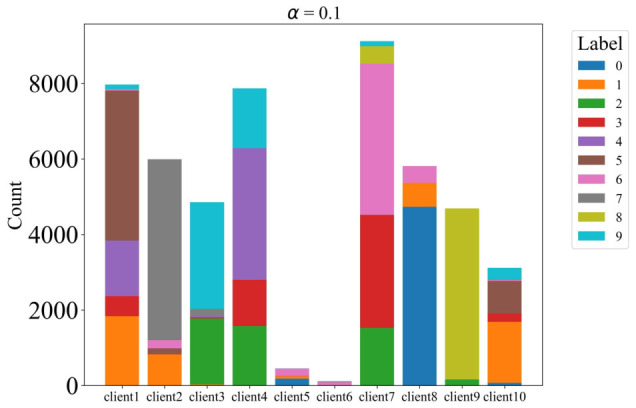
The label distribution when α = 0.1.

**Figure 3:**
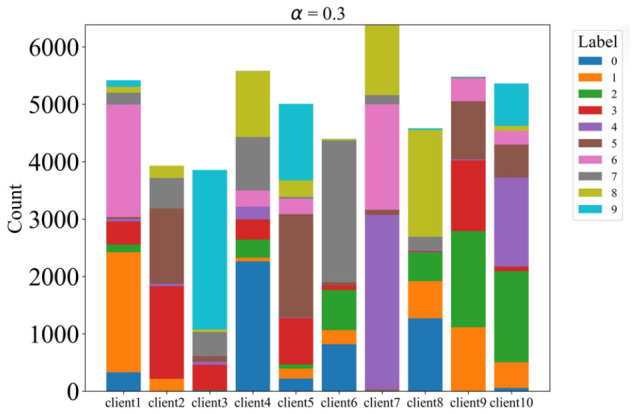
The label distribution when α = 0.3.

**Figure 4:**
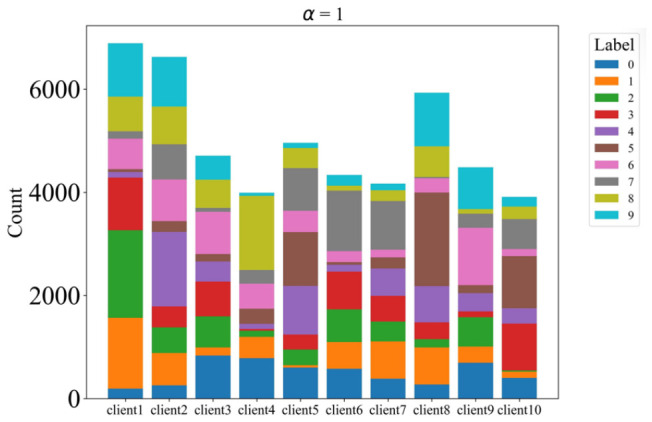
The label distribution when α = 1.

**Figure 5:**
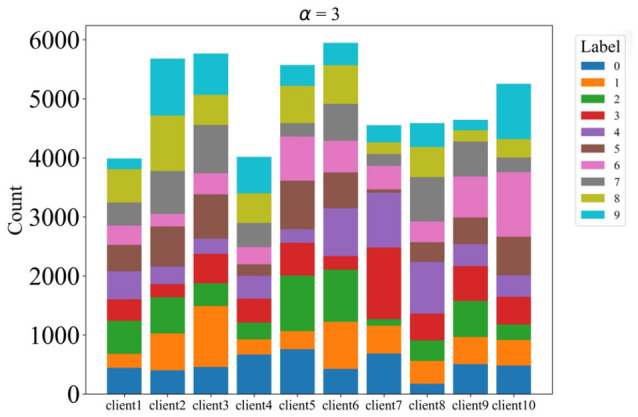
The label distribution when α = 3.

To simulate non-IID data distributions across clients, we partitioned the dataset using a Dirichlet distribution with concentration parameter α, controlling the degree of heterogeneity. We evaluated multiple α values: 0.1, 0.3, 1, and 3, with lower values yielding more heterogeneous distributions. The data is partitioned across 10 clients, and each client performs 5 local training epochs per communication round with a batch size of 64. All clients participate in every round. The detailed distributions are illustrated in Figure 2, Figure 3, Figure 4 and Figure 5.

For the FedAvg baseline, experiments were conducted for each α. For FedAvg with DSSA [22], we additionally varied the server-side sparsity level ($p_{s}=0.3,0.4,0.5,0.6$) and the aggregation ratio ($\eta^{\prime }=3,5,7,10$). Similarly, for the FedProx, experiments were conducted for each α. For FedProx with DSSA experiments, we used the same α, $p_{s}$, and $\eta^{\prime }$ settings, with the proximal term μ further tuned in {0.0005,0.001,0.005,0.01} to enforce local model consistency. We additionally conducted experiments with SCAFFOLD and SCAFFOLD with DSSA under the same parameter settings. All experiments use the Adam optimizer with a fixed learning rate of 0.001 and no learning rate scheduling. 5 seeds were fixed across runs, and no data augmentation was applied.

This experimental setup allows us to systematically evaluate the performance of the proposed FedProx-DSSA method under varying levels of data heterogeneity and sparsity configurations.

In total, the experimental design encompasses multiple combinations of hyperparameters and data heterogeneity levels. Specifically, we conducted:

- 3 FedAvg experiments, each corresponding to a different Dirichlet parameter α;
- 24 FedAvg with DSSA experiments, including all combinations of 3 α values, 2 server-side sparsity levels, and 4 aggregation ratios;
- 3 FedProx experiments, each corresponding to a different Dirichlet parameter α;
- 96 FedProx with DSSA experiments, including all combinations of 3 α values, 2 sparsity levels, 4 aggregation ratios, and 4 proximal term values μ;
- 3 SCAFFOLD experiments, each corresponding to a different Dirichlet parameter α;
- 24 SCAFFOLD with DSSA experiments, including all combinations of 3 α values, 2 sparsity levels, 4 aggregation ratios.

For each hyperparameter configuration, the final-round test accuracy is reported. The effects of different hyperparameters, including data heterogeneity level, server-side sparsity, aggregation ratio, and proximal coefficient, on final performance are systematically examined. Baseline and proposed methods follow the same tuning budget for fair comparison. To ensure statistical reliability, each experimental configuration is repeated five times, and results are reported as the mean ± standard deviation across five runs.

Control groups consist of the standard FedAvg, standard FedProx and standard SCAFFOLD under varying data distributions, as well as the standalone FedAvg with DSSA, FedProx with DSSA and SCAFFOLD with DSSA methods under their respective hyperparameter settings. The final accuracy results are shown in Table 1 and Table 2. This comprehensive design enables a systematic evaluation of the effects of data heterogeneity, sparsity, aggregation strategy, and proximal regularization on federated learning performance.

### 4.2. FedAvg Performance Analysis

**Figure 6:**
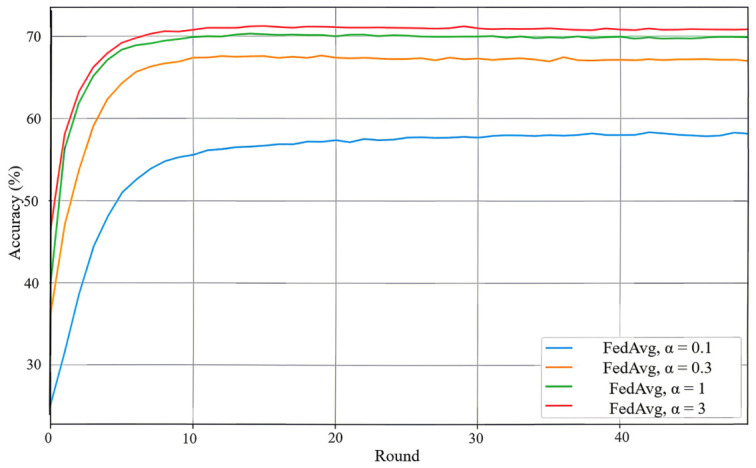
Test accuracy of FedAvg under different data heterogeneity levels (α=0.1,0.3,1,3).

The performance of the standard FedAvg algorithm is strongly affected by the data heterogeneity level controlled by α. As shown in Figure 6, larger α values, which correspond to more homogeneous local data distributions, lead to faster convergence and higher final test accuracy. In particular, when α=3, FedAvg achieves the best performance and reaches more than 72% test accuracy after 50 communication rounds. In contrast, under the highly heterogeneous setting α=0.1, the convergence becomes much slower and the final accuracy drops to around 58%.

This trend is consistent with the well-known client-drift effect in non-IID federated learning. When local data distributions differ significantly across clients, locally optimized models tend to deviate from a common descent direction, which weakens the effectiveness of global aggregation and degrades the final model quality. Therefore, the FedAvg results provide a useful dense-training baseline and further highlight the need for mechanisms that improve robustness under heterogeneous data while controlling resource consumption.

### 4.3. FedAvg with DSSA Performance Analysis

We next evaluate the effect of introducing DSSA into FedAvg. The main purpose of DSSA is to reduce bidirectional communication and local computation through structured sparsification, while preserving as much learning performance as possible. The results show that the performance of FedAvg with DSSA depends strongly on the sparsity level and aggregation ratio. A moderate sparsity configuration generally provides a better trade-off between accuracy and efficiency, whereas overly aggressive sparsification leads to a more noticeable performance loss, especially under heterogeneous data.

**Figure 7:**
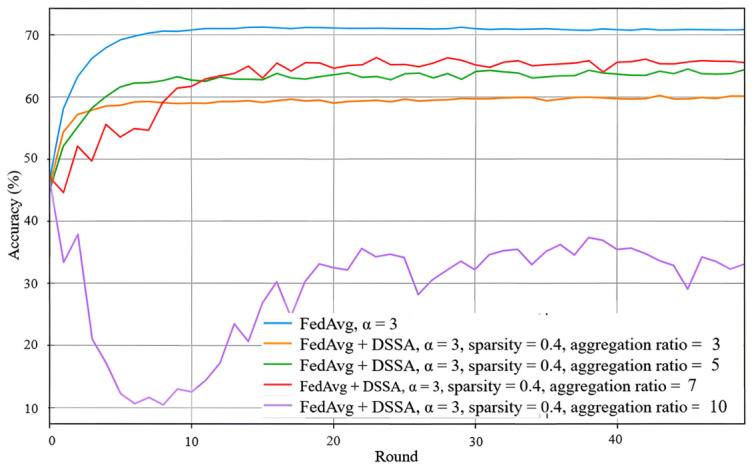
Test accuracy of FedAvg with DSSA under α=3, sparsity p=0.4, and aggregation ratio =3,5,7,10.

As illustrated in Figure 7, when α=3, using sparsity p=0.4 with aggregation ratio 7 yields the best result among the tested FedAvg+DSSA settings, with a final accuracy close to 65%. Although this remains below the dense FedAvg baseline, it indicates that DSSA can retain a substantial portion of the learning capability while significantly reducing resource consumption. Under stronger non-IID conditions, the accuracy degradation becomes more evident, suggesting that sparsification alone is not sufficient to fully counteract the optimization difficulty caused by client heterogeneity.

**Figure 8:**
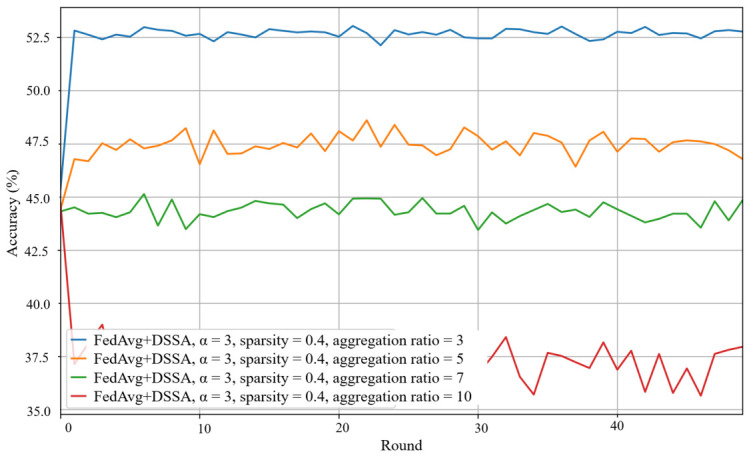
Test accuracy of FedAvg with DSSA with fixed mask under α=3, sparsity p=0.4, and aggregation ratio =3,5,7,10.

**Figure 9:**
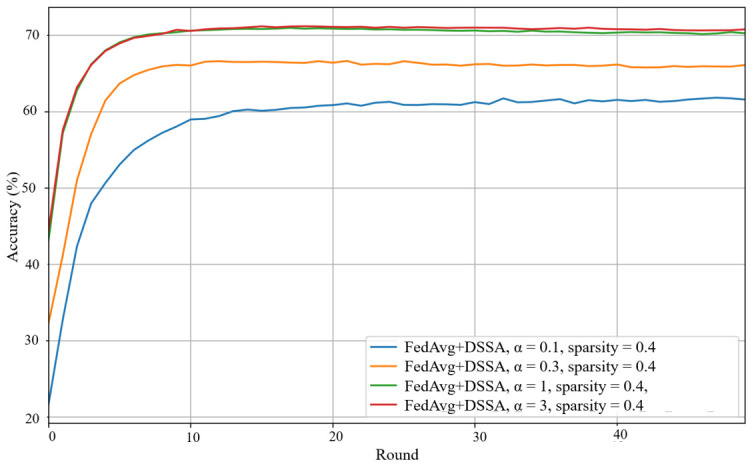
Test accuracy of FedAvg with DSSA with alternative update under α=0.1,0.3,1,3, sparsity p=0.4.

To further understand the design choices of DSSA, we conduct two additional comparative experiments. First, we replace the dynamic mask rotation mechanism with a fixed mask that remains unchanged across all communication rounds after the initial pruning. As illustrated in Figure 8, under the same sparsity level and data heterogeneity, the fixed mask strategy achieves notably lower accuracy than the dynamic mask used in DSSA. This confirms that periodically recomputing the mask based on the current dense model helps different parameters participate in training over time, preventing permanent freezing of the same subset of weights. Second, we compare DSSA with an alternative sparse-updating approach where clients still receive the pruned model but train on the full set of parameters locally, and then upload the full model updates. As illustrated in Figure 9, this alternative achieves slightly higher accuracy than DSSA in some settings. However, it incurs substantially higher overhead: clients perform dense local training, resulting in 100% of the computation cost of standard FedAvg, whereas DSSA updates only the complementary coordinates, thereby reducing local computation proportionally to the sparsity level. Moreover, the uplink communication of this alternative transmits the full model update rather than only the complementary sparse update, eliminating the uplink saving that DSSA provides. Therefore, while the alternative sparse-updating method yields modest accuracy gains, it does so at the expense of both computation and uplink communication efficiency, representing a different point in the accuracy-resource trade-off rather than a strict improvement over DSSA.

Overall, the results indicate that DSSA can provide a favorable accuracy–efficiency trade-off when its hyperparameters are properly selected. Its benefit lies primarily in reducing communication and computation overhead, rather than in improving absolute accuracy over dense FedAvg.

### 4.4. FedProx with DSSA Performance Analysis

We further combine DSSA with FedProx to improve robustness under highly heterogeneous data. In this setting, the proximal coefficient μ plays an important role in balancing local adaptation and global consistency. A relatively small μ provides useful regularization against client drift without excessively restricting local learning, whereas an overly large μ may slow optimization by constraining local updates too strongly.

**Figure 10:**
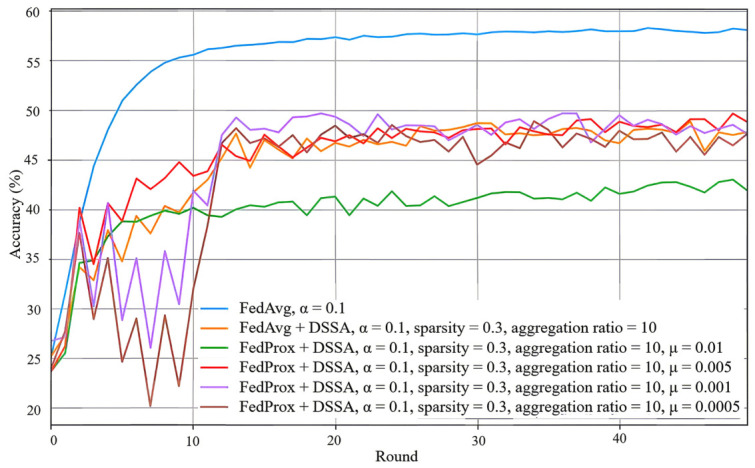
Test accuracy of FedProx with DSSA under α=0.1, sparsity p=0.3, aggregation ratio =10, and μ=0.01,0.005,0.001,0.0005.

Under the challenging setting α=0.1, FedProx with DSSA achieves the best performance among the sparse methods considered in this work. In particular, the configuration with μ=0.005, sparsity p=0.3, and aggregation ratio 10 reaches approximately 48% accuracy. This corresponds to a modest improvement over the corresponding FedAvg with DSSA setting, indicating that proximal regularization can partially alleviate the performance degradation introduced by severe data heterogeneity. As shown in Figure 10, FedProx with DSSA also tends to produce smoother convergence behavior across communication rounds, particularly during the earlier stage of training.

Nevertheless, the sparse methods still remain below the dense FedAvg baseline in absolute accuracy under the same highly non-IID condition. Therefore, the main advantage of FedProx with DSSA is not to surpass dense training, but to offer a more robust sparse alternative that improves the accuracy–efficiency trade-off under heterogeneous data.

We note that while the above results demonstrate the combined effect of dynamic mask rotation, complementary updating, and FedProx integration, the distinct contribution of each individual component has not been independently quantified in our current experimental design. The observed performance improvements arise from the interplay of these mechanisms rather than from any single component in isolation. A full ablation study that separately isolates each component would require additional experiments beyond the scope of this paper, but we acknowledge this as an important direction for future work. Readers should therefore interpret the reported results as evidence of the combined effectiveness of the proposed framework rather than as a component-wise attribution of gains.

### 4.5. Communication and Computational Overhead

The communication and computational overhead results are summarized in Table 3. Compared with dense FedAvg, DSSA consistently reduces both transmitted model size and total computation volume across all tested sparsity levels. The reported computation values represent the total floating-point operations for local training, including forward propagation, backward propagation, and optimizer updates. The reduction primarily comes from the optimizer update step, where only the complementary (pruned) weights are updated while the retained weights remain frozen. Forward and backward propagation remain dense in our current PyTorch 2.1.0 implementation. Specifically, the total computation decreases from 272.0 GFLOPs under FedAvg to 217.5, 204.0, 190.5, and 177.0 GFLOPs at sparsity levels p=0.3, 0.4, 0.5, and 0.6, respectively, corresponding to a maximum reduction of 34.9%. Meanwhile, the communication overhead decreases from 1150.0 MB to 310.0 MB, yielding a maximum reduction of 73.0%.

These savings arise because DSSA reduces the number of parameters transmitted in both downlink and uplink communication, while also reducing the number of parameters that require optimizer updates during local training. The server-side top-*k* pruning and the client-side masking operations introduce only limited additional overhead compared with the cost of repeated local forward and backward propagation.

When DSSA is combined with FedProx, the communication cost remains unchanged, and the extra computational cost introduced by the proximal term is small. For example, at p=0.5, FedProx + DSSA requires 192.5 GFLOPs compared with 190.5 GFLOPs for DSSA alone. This indicates that the robustness benefit of FedProx is obtained with only a minor increase in computation.

Overall, the results show that DSSA achieves substantial communication and computation savings, while FedProx can partially mitigate the associated accuracy degradation under heterogeneous data. This suggests that, within the scope of our experimental setup, the proposed framework provides a favorable resource–performance trade-off for federated learning in edge sensing systems.

## 5. Conclusions

Within the scope of our experimental evaluation, DSSA provides a favorable framework for resource-efficient federated learning under heterogeneous data distributions. By combining the communication and computation benefits of DSSA with the proximal regularization mechanism of FedProx, the proposed method improves the robustness of sparse federated training in non-IID environments. The experimental results show that DSSA can substantially reduce communication overhead and local computation cost in controlled benchmark settings. Although its absolute accuracy may still remain below that of dense FedAvg in some cases, the proposed framework offers a favorable trade-off between learning performance and resource efficiency under the evaluated configurations.

Under the benchmark conditions we evaluated, these properties suggest that DSSA may have potential relevance for communication- and computation-constrained federated learning scenarios, such as smartphone- and wearable-based personalized services, collaborative IoT and edge sensing networks, privacy-preserving healthcare analytics across distributed institutions, and autonomous multi-agent systems that require efficient decentralized model training. However, the current evidence is obtained under controlled benchmark evaluations on CIFAR-10 and SVHN with simulated non-IID partitions. Further validation on real-world edge sensing platforms would be necessary to assess its viability beyond controlled benchmark settings.

We note that the present study is limited to CNN-based image classification benchmarks and does not yet include comparisons with methods such as FedNova or more recent communication-efficient FL baselines. The empirical evidence supports the claimed resource-performance trade-off specifically under the tested CNN architectures on CIFAR-10 and SVHN. Broader generalization to other model architectures, data modalities, and federated optimization algorithms remains an important direction for future work. Overall, within the scope of our experimental setup, the proposed framework demonstrates the potential of combining structured sparsification with proximal regularization to support efficient and privacy-aware learning in resource-constrained sensing environments.

## Figures and Tables

**Table 1:** Test accuracy on CIFAR-10 of FedAvg, FedProx, SCAFFOLD, and their DSSA variants under different data heterogeneity levels (α=0.1,0.3,1), sparsity ratios *p*, and aggregation ratios.

Algorithm	α	*p*	Agg. Ratio	Mean ± Std Acc
FedAvg	0.1	–	–	58.0 ± 1.5%
FedProx	0.1	–	–	62.1 ± 1.7%
SCAFFOLD	0.1	–	–	62.1 ± 2.4%
FedAvg + DSSA	0.1	0.3	10	49.2 ± 1.6%
FedAvg + DSSA	0.1	0.6	5	47.2 ± 1.8%
FedProx + DSSA	0.1	0.3	10	48.7 ± 1.1%
FedProx + DSSA	0.1	0.6	5	47.1 ± 1.8%
SCAFFOLD + DSSA	0.1	0.3	5	51.6 ± 3.1%
SCAFFOLD + DSSA	0.1	0.6	5	49.2 ± 3.4%
FedAvg	0.3	–	–	67.4 ± 1.1%
FedProx	0.3	–	–	68.9 ± 1.3%
SCAFFOLD	0.3	–	–	69.0 ± 1.8%
FedAvg + DSSA	0.3	0.3	10	62.6 ± 1.6%
FedAvg + DSSA	0.3	0.6	5	58.5 ± 1.7%
FedProx + DSSA	0.3	0.3	10	63.5 ± 1.4%
FedProx + DSSA	0.3	0.6	10	59.4 ± 1.7%
SCAFFOLD + DSSA	0.3	0.3	5	64.4 ± 2.3%
SCAFFOLD + DSSA	0.3	0.6	5	61.2 ± 1.9%
FedAvg	1	–	–	70.4 ± 1.2%
FedProx	1	–	–	71.2 ± 1.2%
SCAFFOLD	1	–	–	73.7 ± 0.4%
FedAvg + DSSA	1	0.3	7	67.1 ± 1.4%
FedAvg + DSSA	1	0.6	3	59.4 ± 1.7%
FedProx + DSSA	1	0.3	7	67.0 ± 1.6%
FedProx + DSSA	1	0.6	3	60.9 ± 1.7%
SCAFFOLD + DSSA	1	0.3	3	67.7 ± 0.9%
SCAFFOLD + DSSA	1	0.6	3	65.8 ± 0.8%

**Table 2:** Test accuracy on SVHN of FedAvg, FedProx, SCAFFOLD, and their DSSA variants under different data heterogeneity levels (α=0.1,0.3,1), sparsity ratios *p*, and aggregation ratios.

Algorithm	α	*p*	Agg. Ratio	Mean ± Std Acc
FedAvg	0.1	–	–	86.2 ± 1.3%
FedProx	0.1	–	–	87.1 ± 1.5%
SCAFFOLD	0.1	–	–	88.7 ± 2.8%
FedAvg + DSSA	0.1	0.3	10	65.2 ± 1.4%
FedAvg + DSSA	0.1	0.6	5	63.7 ± 1.4%
FedProx + DSSA	0.1	0.3	10	82.7 ± 1.2%
FedProx + DSSA	0.1	0.6	5	80.1 ± 1.3%
SCAFFOLD + DSSA	0.1	0.3	5	84.7 ± 2.3%
SCAFFOLD + DSSA	0.1	0.6	5	83.2 ± 3.0%
FedAvg	0.3	–	–	89.3 ± 1.4%
FedProx	0.3	–	–	90.2 ± 1.3%
SCAFFOLD	0.3	–	–	90.6 ± 2.1%
FedAvg + DSSA	0.3	0.3	10	76.5 ± 1.5%
FedAvg + DSSA	0.3	0.6	5	75.1 ± 1.4%
FedProx + DSSA	0.3	0.3	10	87.8 ± 1.3%
FedProx + DSSA	0.3	0.6	10	86.7 ± 1.5%
SCAFFOLD + DSSA	0.3	0.3	5	88.5 ± 2.7%
SCAFFOLD + DSSA	0.3	0.6	5	87.9 ± 2.8%
FedAvg	1	–	–	90.1 ± 1.3%
FedProx	1	–	–	91.4 ± 1.4%
SCAFFOLD	1	–	–	92.6 ± 0.3%
FedAvg + DSSA	1	0.3	7	77.8 ± 1.3%
FedAvg + DSSA	1	0.6	5	78.8 ± 1.5%
FedProx + DSSA	1	0.3	7	89.8 ± 1.5%
FedProx + DSSA	1	0.6	3	86.8 ± 1.4%
SCAFFOLD + DSSA	1	0.3	3	90.1 ± 0.8%
SCAFFOLD + DSSA	1	0.6	3	88.8 ± 0.4%

**Table 3:** Comparison of communication and computation overhead.

Method	*p*	Comm. (MB)	Comp. (GFLOPs)
FedAvg	–	1150.0	272.0
DSSA	0.3	425.5	217.5
FedProx + DSSA	0.3	425.5	219.5
DSSA	0.4	371.0	204.0
FedProx + DSSA	0.4	371.0	206.0
DSSA	0.5	337.5	190.5
FedProx + DSSA	0.5	337.5	192.5
DSSA	0.6	310.0	177.0
FedProx + DSSA	0.6	310.0	179.0

## Data Availability

The datasets used in this study are publicly available. CIFAR-10 is available at https://www.cs.toronto.edu/~kriz/cifar.html (accessed on 1 March 2026), and SVHN is available at http://ufldl.stanford.edu/housenumbers/ (accessed on 1 March 2026). The code generated and analyzed during the current study is available from the corresponding author upon reasonable request.
